# Potential antioxidant response to coffee — A matter of genotype?

**DOI:** 10.1016/j.mgene.2014.07.003

**Published:** 2014-08-07

**Authors:** Ute Hassmann, Larisa M. Haupt, Robert A. Smith, Swantje Winkler, Gerhard Bytof, Ingo Lantz, Lyn R. Griffiths, Doris Marko

**Affiliations:** a University of Vienna, Department of Food Chemistry and Toxicology, Währingerstr. 38, 1090 Vienna, Austria; b Genomics Research Centre, Institute of Health and Biomedical Innovation, Queensland University of Technology, Musk Ave, Kelvin Grove, QLD 4059, Australia; c Tchibo GmbH, Überseering 18, D-22297 Hamburg, Germany

**Keywords:** Coffee, Human intervention trial, Genotype, Antioxidant, Nrf2, Chemoprevention

## Abstract

In a human intervention study, coffee combining natural green coffee bean constituents and dark roast products was identified as a genotype-dependent inducer of the Nrf2/ARE pathway, significantly affecting Nrf2 gene expression and downstream GST1A1 and UGT1A1 gene transcription. The observed transcriptional changes correlated with the presence of specific Nrf2 genotypes suggesting their influence on both Nrf2 and subsequent ARE-dependent GST1A1 and UGT1A1 transcription. While the presence of the − 653 SNP seems to be advantageous, resulting in higher Nrf2, GST1A1 and UGT1A1 gene transcription following coffee consumption, in contrast, the presence of the − 651 SNP significantly down-regulated the response to the study coffee. Furthermore, the presence of the B/B genotype in *GST1A1* along with the frequency of the [TA]_6/6_ and [TA]_7/7_ polymorphisms in *UGT1A1* appeared to significantly increase sensitivity toward coffee-induced gene transcription. This data suggests that when examining the role of the Nrf2/ARE pathway in the regulation of antioxidative and chemopreventive phase II efficacy, individual genotypes should be included when considering the potency of bioactive food/food constituents and their therapeutic potential.

## Introduction

Phase II enzymes play a critical role in converting reactive electrophiles or xenobiotics into less toxic products and seem to be crucial for cancer prevention, with deficiency in phase II enzymatic activity previously associated with increased colon cancer risk (Prochaska et al., 1992, Talalay et al., 1988). The expression of many phase II genes is regulated via the activation of antioxidant response elements (ARE), located in the 5′-flanking region of their respective promoters. Activation by ROS or upstream protein kinases induces translocation of the transcription factor Nrf2 (Nuclear factor-erythroid 2 p45 subunit (NF-E2)-related factor 2) from the cytoplasm into the nucleus, binding to ARE and subsequent transcription of phase II enzymes, including glutathione *S*-transferases (GST), UDP-glucuronyl transferases (UGT) or γ-glutamyl cysteine ligase (γGCL) (Balogun et al., 2003, Chen et al., 2004, Ishii et al., 2000, Itoh et al., 1997, Kensler et al., 2007, Kwak et al., 2002, Li et al., 2005, McMahon et al., 2001, Rushmore et al., 1991, Yan et al., 2005). Nuclear translocation is the key to the onset of ARE-dependent phase II gene transcription, however, Nrf2 itself possesses an ARE-like sequence and Nrf2 gene transcription can also be activated through this system (Kwak et al., 2002). Several coffee constituents have already been reported to act as inducers of the Nrf2 pathway, including the diterpenes kahweol and cafestol (Cavin et al., 2002), as yet these appear to play a minor role in filtered coffee (Gross et al., 1997). During a human intervention trial, we recently, identified the typical dark roast coffee constituent *N*-methylpyridinium (NMP) as well as the green bean constituent n-chlorogenic acid (CGA), as potent activators of the Nrf2/ARE pathway (Boettler et al., 2011). Over four weeks, participants consumed the study coffee, a blend combining both these constituents (typical dark roast coffee constituents as NMP with considerable amounts of green bean constituents) resulting in a significant increase in mean Nrf2 gene transcripts in peripheral blood lymphocytes (PBLs) (Boettler et al., 2012, Volz, 2010). In addition, large differences in Nrf2 activation ability were identified in participants, which could not be explained by differences in their general health status or nutritional behavior.

A growing understanding of the complex interactions between genotype, diet, lifestyle, and the environment has already influenced clinical medicine toward more personalized treatments which include the analysis of individual genotype and phenotype giving rise to personalized nutrition recommendations and medical interventions (Go et al., 2004). Applying the same parameters, nutritional genomics, an emerging field of nutritional science, examines genome-wide influences of nutrition and its subsequent role on diagnostic and therapeutic options. Recently, the contribution of nutrition to disease has been recognized to be as important as environmental factors e.g. cigarette smoking and are thought to account for at least 50% of disease risk (DeBusk et al., 2005, McGinnis and Foege, 1993, Zeisel et al., 2005). Thus, nutritional genomics may have important potential toward the diagnosis and prevention of diet-related diseases.

In the Nrf2/ARE pathway, the previous reports have questioned the contribution of genetic variation in specific ARE-dependent antioxidative genes in activation by bioactive food compounds (Ferguson, 2009). In addition, biochemical studies have associated allelic variations in the Nrf2 promoter region with differences in antioxidative phase II gene transcription (Marzec et al., 2007). To date, 9 single nucleotide polymorphisms (SNPs) have been identified in the Nrf2 gene (Marzec et al., 2007, Siedlinski et al., 2009, Yamamoto et al., 2004). Of relevance to this study are the − 617C/A and the − 651G/A SNPs, located within the promoter region. Both SNPs have been shown to reduce Nrf2 transcriptional activity reflected by attenuated binding of Nrf2 to the ARE, resulting in decreased Nrf2 transcription (Marzec et al., 2007). Genotyping a subset of individuals from our previous study indicated a connection between Nrf2 genotype and response to coffee (Boettler et al., 2012). Based on these findings, we undertook the current larger pilot intervention trial, utilizing a special study coffee blend, combing typical dark roast coffee constituents with average amounts of green bean constituents to: (a) test Nrf2-activation potential of the study coffee in a larger cohort than (Boettler et al., 2012); and (b) to determine whether individual differences in response to the study coffee was associated with specific genotypes in Nrf2, GST1A1 and UGT1A1.

## Results

### Nrf2 genotype and gene transcription

A 424 bp polymorphic region of the *Nrf2* promoter was sequenced in all participants (n = 49) who followed an 8 week-coffee intervention trial (for details see Experimental section) to examine the Nrf2 − 617C/A, − 653A/G and the − 651G/A polymorphisms, previously determined to be functionally relevant for transcriptional activity of the Nrf2 gene (Marzec et al., 2007, Yamamoto et al., 2004). The − 617C/A SNP was present in 10/49 (20%) of participants (Table 1). The presence of the − 651G/A SNP was observed in 15/49 individuals (31%) while 31/49 (63%) of the participants possessed a SNP at position − 653A/G. Changes in Nrf2 gene transcription were also assessed at the different blood collection (BC) points during the course of the study.

**Fig. 1:**
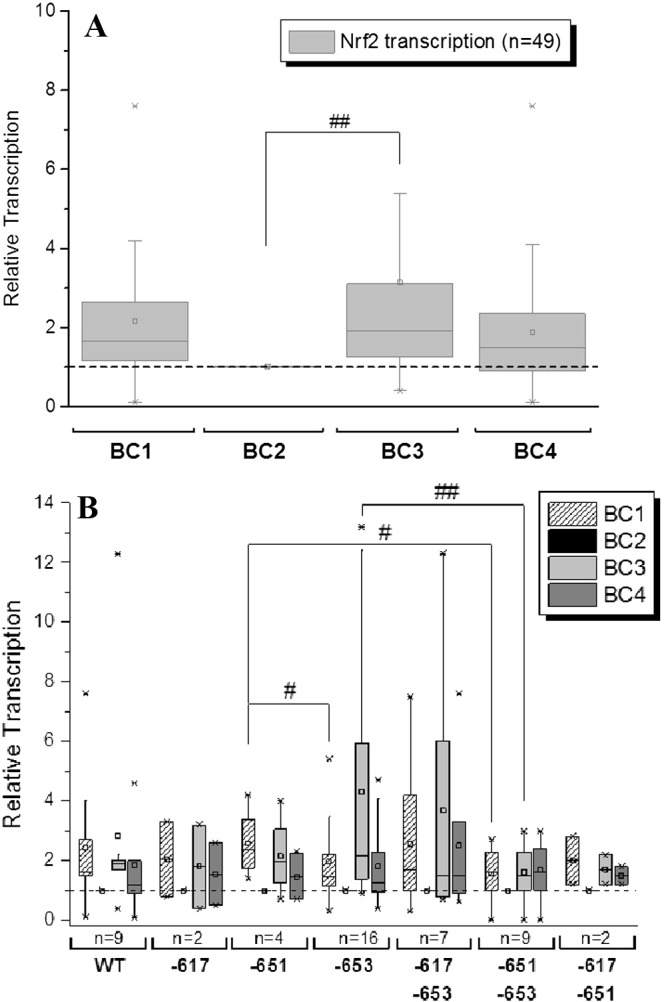
(A) Modulation of Nrf2 gene transcription in PBLs of 49 participants of the intervention trial (samples of two participants failed to show sufficient fluorescence signal/amplification). The data, analyzed in triplicate are presented as BOX plots, normalized to ß-actin expression and as relative transcription of individual levels of each participant before the study (BC1), after a four week wash-out (polyphenol-poor diet and no coffee consumption, BC2), after four weeks of daily 750 mL coffee consumption (BC3), and after a second wash-out period of four weeks (BC4). The data are represented as relative transcription of BC2 = 1. Significances indicated were calculated by Student's t-test (#p < 0.01) on the basis of the relative expression values. (B) Nrf2 transcription according to the Nrf2 polymorphisms − 617, − 651, − 653, − 617 + − 653, − 651 + − 653 and − 617 + − 651 during the human intervention study. Depicted data are relative transcripts normalized on ß-actin as endogenous control and in relation to BC2. Significances indicated are calculated on basis of relative expression values BC1: − 651 to − 653 and − 651 + − 653; BC3: − 652 to − 651 + − 653 (Wilcoxon rank sum test #p < 0.05 and ##p < 0.01). BC: blood collection; WT: wild type.

A significant induction of Nrf2 gene transcription after four weeks consumption of the study coffee (BC3) in comparison to the first wash-out timepoint (BC2) was demonstrated. Nrf2 gene transcription was also elevated under normal nutrition conditions (BC1) in comparison to BC2, however not significantly (Fig. 1A). In total, 65% (32/49) of participants displayed a ≥ 1.5-fold induction of Nrf2 gene transcription following coffee consumption, indicating a functionally significant response in terms of antioxidative gene activation in healthy individuals.

When we more closely examined the level of genotype-dependent Nrf2 activation, clear genotype-dependent activation patterns became apparent. Individuals possessing the − 651 SNP demonstrated a significantly higher activation level at the normal nutrition BC1 timepoint than subjects possessing the − 653 SNP (*p* = 0.026) (Fig. 1B). Specifically in responders (≥ 1.5-fold increase), 12/16 (75%) of − 651 carriers demonstrated a significantly elevated Nrf2 gene transcription at BC1 with 8/16 (50%) of − 653 SNP carriers showing this increase. The presence of the − 653 SNP in participants with reduced the susceptibility of Nrf2 activation through normal nutrition (BC1) was further reflected by the observed significantly higher Nrf2 activation of homozygous − 651 SNP carriers when compared with heterozygous − 651 + − 653 SNP carriers (*p* = 0.017) along with reduced frequency (− 651 + − 653 SNP carriers: 5/9 (55%)) (Fig. 1B).

In contrast, at the BC3 timepoint, solo − 653 SNP carriers showed a significantly higher increase in Nrf2-transcription than those, who carried the − 651SNP, as well (*p* = 0.011). This was also demonstrated in the frequency of activation with 11/16 (69%) of solo − 653 SNP carriers demonstrating significant Nrf2 activation following coffee consumption compared to 5/9 (55%) of − 651 + − 653 SNP carriers, suggesting the observed diet-dependent activation of Nrf2 transcription is associated with Nrf2-genotype.

### GST1A1 genotype and gene transcription

**Fig. 2:**
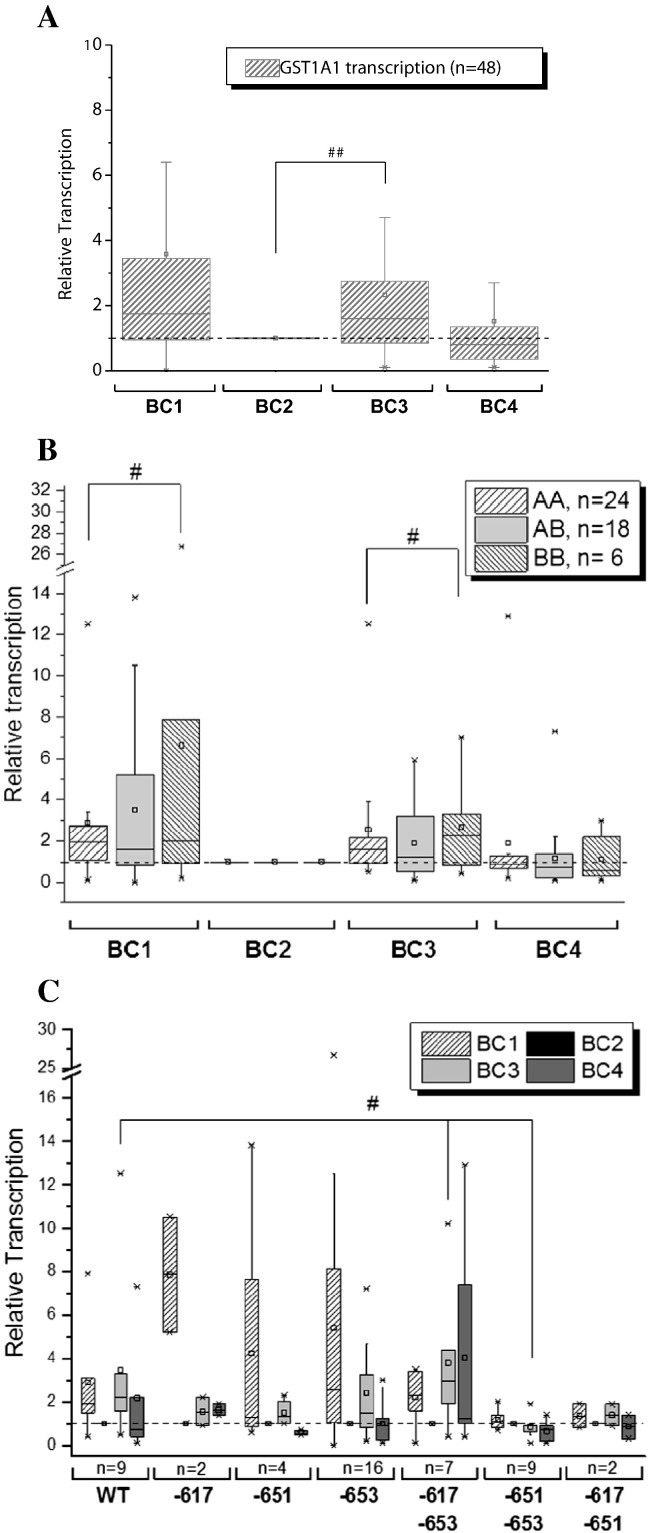
(A) Modulation of the GST1A1 gene transcription in PBLs of 48 participants of the intervention trial (samples of three participants failed to show sufficient fluorescence signal/amplification). The data, performed in triplicate are presented as BOX plot, normalized to ß-actin expression and as relative transcription of individual levels of each participant before the study (BC1), after a four-week wash-out (polyphenol-poor diet and no coffee consumption, BC2), after four weeks of daily 750 mL coffee consumption (BC3), and after a second wash-out period of four weeks (BC4). The data are represented as relative transcription of BC2 = 1. Significances indicated were calculated by Student's t-test (##p < 0.01) on the basis of relative expression values. (B) GST1A1 transcription according to the GST1A1 polymorphisms A/A, A/B and B/B during the human intervention study. Depicted data are relative transcripts normalized on ß-actin as endogenous control and in relation to BC2. Significances indicated BC1: A/A to B/B, BC3: A/A to B/B (C) GST1A1 transcription according to the Nrf2 polymorphisms − 617, − 651, − 653, − 617 + − 653, − 651 + − 653 and − 617 + − 651 during the human intervention study Depicted data are relative transcripts normalized ß-actin as endogenous control and in relation to BC2. Significances indicated, calculated on basis of relative expression values (BC3–BC2): − 617 + − 653 to WT and − 651 + − 653 (Wilcoxon rank sum test #p < 0.05).BC: blood collection; WT: wild type.

We next examined the GST1A1 polymorphism comprised of the two alleles, GST1A1*A and GST1A1*B (Coles et al., 2001). The two genotypes differ in three linked base changes in the 5′-regulatory region of the gene (a T/G change at − 567, a C/T change at − 69 and G/A change at − 52). From the intervention trial, 24/48 (50%) of participants were found to carry the A/A genotype, 18/48 (38%) the heterozygous A/B genotype and 6/48 (12%) the rare homozygous B/B genotype (Table 1). We then investigated whether four weeks of intervention with the study coffee modulated GST1A1 transcription levels, an important member of the antioxidative GST enzyme family. GST1A1 gene transcription was potently elevated in the majority of participants after four weeks consumption of the study coffee (BC3) in comparison to the wash-out phase (BC2, Fig. 2A). Stratification of participants with respect to coffee response demonstrated that 24/49 (49%) of study participants possessed a ≥ 1.5-fold induction of GST1A1 transcription following coffee consumption. Furthermore, this group also displayed a higher induction of GST1A1 transcription during the BC1 phase of the study where participants followed their normal individual nutritional diet. When we then compared the GST1A1 genotype in relation to changes in GST1A1 transcription, significant differences in GST1A1 activation potential were evident. AA carriers appear to possess a lower activation potential than BB carriers irrespective of individual nutrition (BC1, *p* = 0.031) first wash-out (BC2, *p* = 0.018) or consumption of the intervention coffee at BC3 (*p* = 0.018) (Fig. 2B).

The GST1A1 gene carries an ARE sequence in its promoter region, hence its transcription is activated by Nrf2. Our data demonstrates the transcriptional activity of Nrf2 and subsequently the ability to activate *GST1A1* as an ARE-dependent gene appears to be dependent on the specific Nrf2 genotype of the individual following consumption of the study coffee but not during normal nutrition (Fig. 2C). Subjects carrying the − 651 + − 653 Nrf2 SNP showed no activation of GST1A1 gene transcription following coffee consumption (BC3) in comparison to the preliminary wash-out period (BC2). This lack of *GST1A1* activation ability of − 651 + − 653 carriers after coffee consumption significantly became apparent when compared to WT (*p* = 0.017) and − 617 + − 653 SNP (*p* = 0.039) carriers. This difference is further evident when the allelic frequency of coffee responders (≥ 1.5-fold increase) is examined. Whereas 7/9 (78%) of WT carriers and 5/7 (71%) of − 617 + − 653 SNP carriers, respectively displayed significant increases in Nrf2 gene transcription after coffee consumption, only 2/9 (22%) of subjects carrying − 651 + − 653 SNP could be identified as coffee responders. This supports our observations that the presence of the second SNP at position − 651 SNP appears to reduce coffee-induced Nrf2 activation (Fig. 2C).

**Fig. 3:**
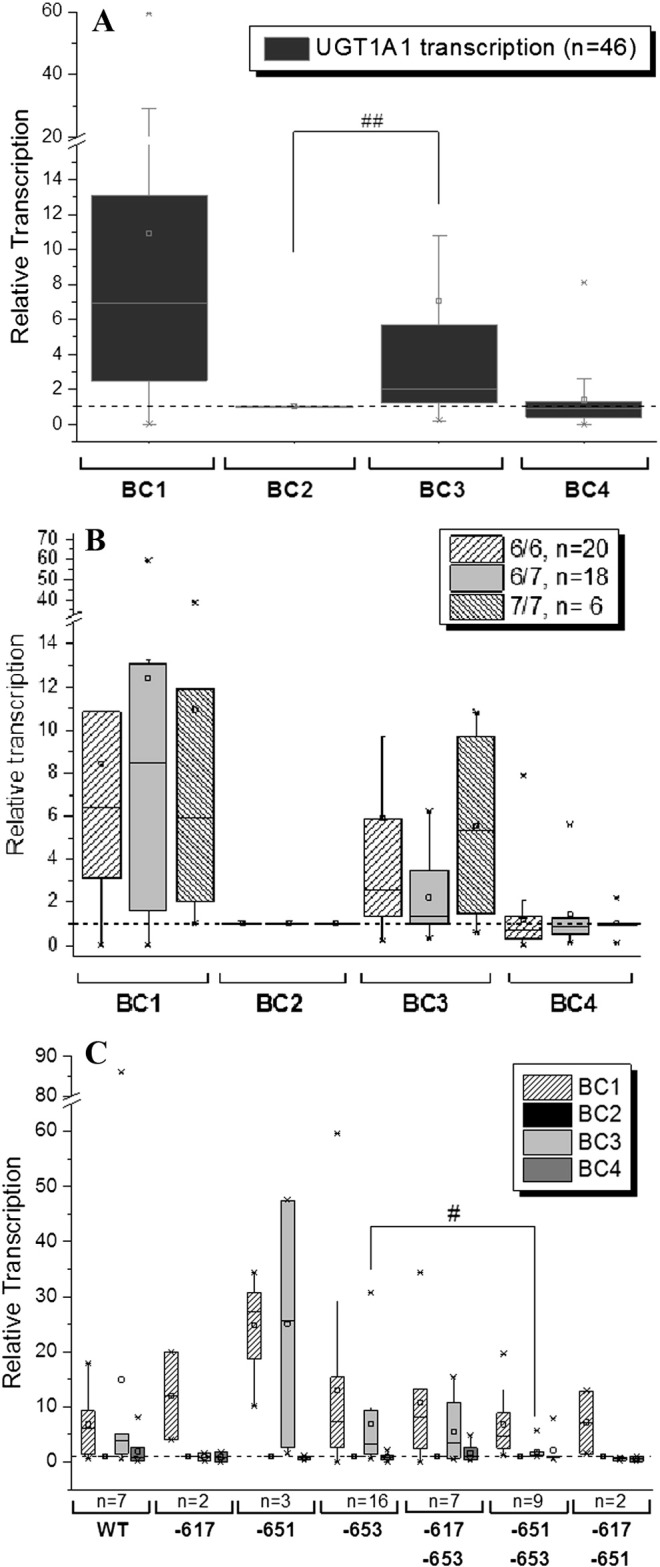
(A) Modulation of the UGT1A1 gene transcription in PBLs of 46 participants of the HSG trial (samples of five participants failed to show sufficient fluorescence signal/amplification). The data, performed in triplicate are presented as BOX plot, normalized to ß-actin expression and as relative transcription of individual levels of each participant before the study (BC1), after a four week wash-out (polyphenol-poor diet and no coffee consumption, BC2), after four weeks of daily 750 mL coffee consumption (BC3), and after a second wash-out period of four weeks (BC4). The data are represented as relative transcription of BC2 = 1. BC: blood collection. Significances indicated were calculated by Student's t-test (##p < 0.01), on the basis of relative expression values. (B) UGT1A1 transcription according to the UGT1A1 polymorphisms [TA]_6/6_, [TA]_6/7_ and [TA]_7/7_ during the human intervention study. Depicted data are relative transcripts normalized on ß-actin as endogenous control and in relation to BC2. (C) UGT1A1 transcription according the Nrf2 polymorphisms − 617, − 651, − 653, − 617 + − 653, − 651 + − 653 and − 617 + − 651 during the human intervention study Depicted data are relative transcripts normalized ß-actin as endogenous control and in relation to BC2. Significances indicated, calculated on basis of relative expression values (BC3–BC2): − 653 to − 651 + − 653 (Wilcoxon rank sum test #p < 0.05).BC: blood collection; WT: wild type. BC: blood collection.

UGT1A1 represents another important phase II gene in cellular detoxification processes. With activation of UGT1A1 gene transcription previously only reported in a murine model (Kalthoff et al., 2010), we next examined changes of UGT1A1 gene transcription in the study participants. UGT1A1 possesses a known variation in its promoter sequence (Monaghan et al., 1996), with an additional TA repeat in the TATA region (7 TA repeats; UGT1A1*28) known to markedly decrease UGT1A1 expression resulting in reduced activity (Monaghan et al., 1996, Van der Logt et al., 2004). Most participants of the present intervention trial were found to possess either a homozygote [TA]_6/6_-repeat sequence (wild type, 21/46 individuals (46%)) or a heterozygote ([TA]_6/7_-repeat sequence (19/46 individuals (41%)). Only 13% (6/46) of the individuals were identified as homozygous [TA]_7/7_-repeat sequence carriers (Table 1). Although a wide variation in UGT1A1 transcription levels among participants was detected at the early timepoint (BC1, Fig. 3A), the affect on transcription in response to coffee following adherence to the trial conditions was more evident at the BC3 stage of the trial. On average, a significant induction of UGT1A1 gene transcription was observed in participants after four weeks intervention with the study coffee. Furthermore, the absence of coffee during the second wash-out period (BC4) resulted in down-regulation of UGT1A1 gene expression to basal levels, emphasizing that the study coffee constituents were crucial to the induced expression observed at BC3. When we stratified the participants into responders (BC3 ≥ 1.5 fold increase) and non-responders (BC3 < 1.5 fold increase), 27/44 (61%) of individuals were identified as coffee responders, supporting our previous findings of UGT1A1 gene transcription activation by the study coffee. Although UGT1A1 transcription has been previously defined in a murine model, this is the first time this has been demonstrated in humans (Fig. 3A).

We then correlated changes in UGT1A1 transcription at the different BC points with participant UGT1A1 genotype. This suggested correlation between reduced response to coffee (BC3) and a heterozygous [TA]_6/7_ genotype (Fig. 3B). Individuals, carrying either the homozygous [TA]_6/6_ (*p* = 0.21) or the heterozygous [TA]_7/7_ (*p* = 0.10) genotype responded equally to the study coffee when compared to individuals possessing the [TA]_6/7_ genotype. When we examined UGT1A1 responders (BC3 ≥ 1.5-fold increase), the data further supported this observation with 14/20 (70%) of [TA]_6/6_ carriers and 5/6 (85%) of [TA]_7/7_ carriers responding to the study coffee compared to only 8/18 (44%) of [TA]_6/7_ carriers. However, once again, a genotype specific response became apparent at the BC3 timepoint. Individual nutrition with different sources of polyphenols (BC1) did not demonstrate genotypic dependent activation (frequency of responders: [TA]_6/6_ 17/20 (85%); 6/7 15/18 (83%) and 7/7 5/6 (83%). This may be attributed to the broad range of gene transcription activation observed at the BC1 timepoint when compared to BC3, and is also demonstrated by the observed lower SD.

Correlating UGT1A1 gene transcription to Nrf2 genotype again demonstrated a diet-specific activation pattern. Although no change in UGT1A1 gene transcription was observed among the different Nrf2 genotypes at the BC1 timepoint, following the consumption of the study coffee (BC3), clear differences in gene expression were observed (Fig. 3C). While individuals possessing the WT, the − 651, the − 653 and the − 617 + − 653 SNP showed activation of UGT1A1 gene transcription following coffee consumption (BC3), individuals possessing the − 617, the − 617 + − 651 or the − 651 + 653 SNP displayed no change when compared to the preliminary wash-out period (BC2). When we examined the data more closely, individuals possessing the − 651 + − 653 SNP genotype, again demonstrated significantly reduced activation potential when compared to solo − 653 carriers (*p* = 0.021). When considering the number of UGT1A1 coffee responders (BC3 ≥ 1.5-fold increase) 12/16 (75%) − 653 SNP carriers were identified as coffee responders compared to only 3/9 (33%) of − 651 + − 653 SNP carriers. This effect appears to be dependent of nutritional influence as indicated by the observed expression at the BC1 timepoint with the three genotype groups responding equally (− 651 3/3 (100%); − 653 13/16 (81%) and − 651 + 653 (89%).

## Discussion

Here, we applied a nutrigenomic approach to examine the antioxidative potential of activation of the Nrf2/ARE pathway in response to a study coffee during an intervention trial at three key timepoints. The study coffee was the only polyphenol source (BC3), compared to a wash-out period with a strong reduction of polyphenol uptake (BC2) and individual balanced nutrition timepoint (BC1), with differed sources of polyphenols. To determine, whether the genetic background, specifically the presence of single nucleotide polymorphisms (SNPs), plays a role in the activation potential of the Nrf2 pathway, participant genotypes were assessed.

We identified the study coffee as a potent inducer of the antioxidative and chemopreventive Nrf2/ARE pathway in humans. Significant increases in Nrf2, GST1A1 and UGT1A1 gene transcription were observed after four weeks of coffee consumption in the mean of the 51 participants. Although this trend was clear, notable differences in individual responses to the study coffee (Figs. 1A, 2A, 3A) were also observed. Examining this data more closely revealed a significant (≥ 1.5-fold) increase in Nrf2 (32/49 (65%)), GST1A1 (24/49 (49%)) and UGT1A1 (27/44 (61%) gene transcription following coffee intervention (BC3) when compared to the wash-out period (BC2). The differences in gene expression in response to the study coffee could not be explained by differences in dietary uptake of antioxidants by participants through their general diet, specific foods consumed, coffee intake, gender, ethnicity or age. However, differences in Nrf2 activation potential were observed at the BC1 timepoint, as well as following no restrictions on participant general diet, specific foods consumed or coffee intake. This suggested some contribution of dietary antioxidant uptake to the activation of these genes with Nrf2 (29/49 (59%)) and GST1A1 (30/49 (61%) significantly increased (Figs. 1A, 2A). However, no significant difference between the two groups during normal individual nutrition (BC1) was detected for UGT1A1 gene transcription, with 37/44 (84%) of individuals identified as responders indicating high activation potential of UGT1A1 following the consumption of polyphenols, deriving from different food sources. In order to elucidate any genotypic involvement, i.e. the presence of specific alleles influencing response to coffee, we examined regions within the Nrf2, GST1A1 and UGT1A1 genes, carrying known polymorphisms previously demonstrated to affect the transcriptional activity of these genes (Boettler et al., 2012, Coles et al., 2001, Monaghan et al., 1996, Van der Logt et al., 2004).

Allele frequencies for the − 617 and the − 651 Nrf2 SNPs examined were in concordance with current literature and available data (Table 1). 31/49 (63%) of the participants displayed a SNP at position − 653A/G. Thus, data from this study demonstrated a higher incidence of the − 653 SNP in the Nrf2 promoter among the study cohort. This is in line with previously published data (Boettler et al., 2012), but is higher than data published by Marzec et al. (2007). However, when we combine data from the current study (51 individuals) with the two previous studies (n = 20 in Marzec et al. (2007) and n = 18 in (Boettler et al., 2012)) incidence of the − 653 SNP is 56–63% and within the normal reported HapMap frequency in Caucasians.

We next examined a known polymorphism in the GST1A1 gene (Marzec et al., 2007). The frequency of the GST1A1 genotypes (Table 1) was found to be comparable to other studies in Caucasians (Coles et al., 2001) indicating the study data appears to represent an average population profile.

The UGT1A1 gene possesses the known variation UGT1A1*28 the incidence of which varies among ethnicities, being highest in the African (43%) or European (54%) descended individuals and lowest in those of Asian (16%) descent (Van der Logt et al., 2004). The study population displayed a ratio of genotypes within the expected range (Table 1) and in line with the previous data (Monaghan et al., 1996, Van der Logt et al., 2004).

When we then analyzed the changes in gene transcription of Nrf2, GST1A1 and UGT1A1 during the course of the two-month trial with respect to individual genotype, several associations were revealed. Of note, the genotype-specific response at the individual nutrition timepoint (BC1) and following the coffee intervention period with the study coffee as the only source of polyphenols (BC3) differed significantly. Individuals possessing the − 651 SNP demonstrated significantly higher transcriptional activation of Nrf2 than subjects possessing the − 653 SNP or the − 651 + − 653 SNP at the same time at BC1, also apparent when comparing actual numbers (− 651 SNP carriers 75%, − 653 SNP carriers 50% and − 651 + − 653 55%).

Yet, − 653 SNP carriers showed a significantly higher increase in Nrf2 transcription following coffee consumption (BC3) than those who carried the − 651 SNP, as well (69% of − 653 SNP carriers and 55% of − 651 + − 653 SNP carriers). It appears that the presence of the − 653 SNPs is disadvantageous for Nrf2 gene activation under normal nutrition conditions, however individuals carrying this SNP seem still capable of benefitting from the antioxidative effects of the study coffee, with significantly increased Nrf2 gene transcription (Fig. 2B).

As yet, why the study coffee, as the only polyphenol source during the intervention period, is more capable of activating Nrf2 gene transcription has to be elucidated. During Nrf2 activation, cytosolic Nrf2 must be released from its cytosolic partner Keap1. This release is initiated through oxidative and electrophilic signaling pathways or through direct interaction with the thiol residues of Keap1 (Chen et al., 2004, Kensler et al., 2007, McMahon et al., 2001, Rushmore et al., 1991). Polymorphic genetic variations (SNPs) may potentially alter the structure of the encoding protein, disrupting this initiation (Worth et al., 2007). One might speculate that an altered Nrf2 protein as a result of the SNP at position − 653 might tighten the binding of Nrf2 to Keap1, reducing Nrf2 translocation under normal nutritional conditions. In contrast, the study coffee, due to its high concentration of Keap1-reactive residues is able to interfere with or loosen Nrf2-Keap1 binding. Alternatively, the study coffee may, per sé possess a higher antioxidative potential than normal nutrition thereby overcoming any mediating effects due to the presence of the − 653 SNP. Independently from the source of polyphenols, the presence of the − 651 SNP together with the − 653 SNP seems to be disadvantageous, since − 651 + − 653 SNP carriers reflected the lowest activation potential at both BC1 and BC3 (Fig. 1B). However, significant mechanistic investigations are necessary to fully investigate any diet-dependent associated Nrf2 activation potential in relation to specific SNP genotype.

We next compared the GST1A1 genotype to any associated changes in GST1A1 transcription. A significantly decreased activation potential of AA carriers was observed following to the individual nutrition (BC1), first wash-out (BC2) and study-coffee diet (BC3) timepoints (Fig. 2B). The polymorphism investigated has previously been shown to alter *GST1A1* gene expression (Coles et al., 2001). Using a luciferase reporter assay, hGSTA1 promoters showed differential expression when transfected into HepG2, GLC4 and Caco-2 cells associated with the BB genotype, resulting in lower transcriptional activation (Monaghan et al., 1996). This is in contrast to our data and may be due the use of in vitro models or to the different cell types examined. In addition, although the observed B/B allele frequency is within the expected values for the Caucasians, the relatively low number (6/48) of individuals identified in the study is insufficient to fully determine the significance of this difference. However, this variability in response, suggesting that BB carriers may benefit from functional antioxidative food is worthy of additional follow-up in larger cohorts and mechanistic models to determine its validity.

As *GST1A1* carries an ARE sequence in its promoter region and is able to be activated by Nrf2, to more fully interpret the transcriptional changes of GST1A1 during the course of the intervention trial, it needs to be correlated with Nrf2 genotype. A specific Nrf2 genotype dependent response became apparent after the consumption of the study coffee. Subjects possessing the − 651 + − 653 Nrf2 genotype showed significant activation of GST1A1 gene transcription at BC3 in comparison to the BC2 wash-out timepoint when compared to WT or − 617 + − 653 SNP carriers (Fig. 2B). We confirmed this observation when we examined the effects in terms of responders at BC3 (WT 78%, − 617 + − 653 SNP carriers 71% and − 651 + − 653 SNP carriers 22%). Thus, the presence of the − 651 SNP appears to suppress coffee-induced GST1A1 gene transcription, similar to our observations for Nrf2. The − 651 SNP has previously been shown to reduce transcriptional activity of Nrf2 and to increase the risk of ALI (Marzec et al., 2007), supporting our data. However, this is the first study to report diet-specific activation of downstream Nrf2-dependent genes in association with Nrf2 genotype. Of note, no significant difference between Nrf2 genotype was demonstrated after individual normal nutrition with various sources of polyphenols (BC1).

UGT1A1 gene transcription was also significantly up-regulated following coffee consumption. To better elucidate individual response to the study coffee, transcriptional activity of UGT1A1 was correlated with UGT1A1 genotype. The UGT1A1 gene possesses a (TA)_n_ repeat polymorphism in the TATA-Box. Individuals who carry the [TA]_7/7_ genotype have been shown to have higher bilirubin blood levels and a higher prevalence of Gilbert's syndrome (Siedlinski et al., 2009). Investigating a potential diet-dependent response in relation to genotype demonstrated reduced response to the study coffee (BC3) of individuals carrying the [TA]_6/7_ heterozygous genotype ([TA]_6/7_ carriers 44%, [TA]_6/6_, 70% [TA]_7/7_, 85% carriers; Fig. 3B). No genotype specific response was observed during individual nutrition with different sources of polyphenols at BC1. The [TA]_6/7_ genotype appears to contribute as a blocker of diet-induced gene transcription but does not correlate to Gilbert's syndrome, whereas the [TA]_7/7_ genotype does not appear to play a role in diet-induced antioxidative UGT1A1 transcription but in the prevalence for Gilbert’s syndrome (Monaghan et al., 1996). Further demonstrating the complexity of genotype and gene-function interactions.

As the only polyphenol source, the study coffee demonstrated Nrf2 genotype-dependency with respect to UGT1A1 gene transcription (Fig. 3C). Individuals possessing, either a WT, a − 651, a − 653 or a − 617 + − 653 genotype displayed increased UGT1A1 gene transcription at BC3, whereas individuals possessing the − 617, the − 617 + − 651 or the − 651 + 653 SNP showed no effect. When we focused on the − 653 SNP individual's carrying the − 651 + − 653 SNP genotype displayed significantly reduced UGT1A1 gene transcription when compared to solo − 653 carriers (− 653 SNP 75% and − 651 + − 653 SNP carriers 33%). Once again, these results indicate that the presence of the second SNP at position − 651 may modify the diet-dependent transcriptional response. However, no such observation was shown at BC1 with all three genotypes equally responsive (− 651 100%, − 653 81% and − 651 + 653 89%), supporting a genotypic diet-dependency on gene expression. Overall, the findings of this study demonstrate the influence of genotype on general/physiological gene transcription. We have expanded this to examine the specific effect of Nrf2 genotype on transcriptional activation following food intervention.

## Conclusion

Taken together, the study coffee, combining potent Nrf2 inducers from green coffee beans with typical high roasted constituents, has been identified as a potent inducer of antioxidative gene transcription, most likely resulting in higher enzymatic activity in participants in a two-month coffee intervention trial. Differences in the coffee response within the study population resulted in the identification of a group of responders and non-responders for the transcription of Nrf2, GST1A1 and UGT1A1 in peripheral blood lymphocytes. Correlation altered gene transcription with genotype indicated that the Nrf2 genotype appears to influence its own transcription as well as the downstream ARE-dependent genes GST1A1 and UGT1A1. While the presence of the − 653 SNP seems to increase sensitivity to the antioxidative properties of the study coffee, resulting in a higher transcription of Nrf2, GST1A1 and UGT1A1, the coincidently presence of the − 651 SNP significantly down-regulated individual's ability to respond to the study coffee for the three genes investigated. Of note, these effects seem appear to be strongly influenced by diet with these genotype-dependent differences not detected under normal nutrition conditions, potentially indicating a substantial role of polyphenol source with antioxidative gene activation. In addition, the presence of the B/B genotype in *GST1A1* as well as the frequency of either [TA]_6/6_ or [TA]_7/7_ in *UGT1A1* appears to increase sensitivity toward coffee-induced transcription.

**Fig. 4:**
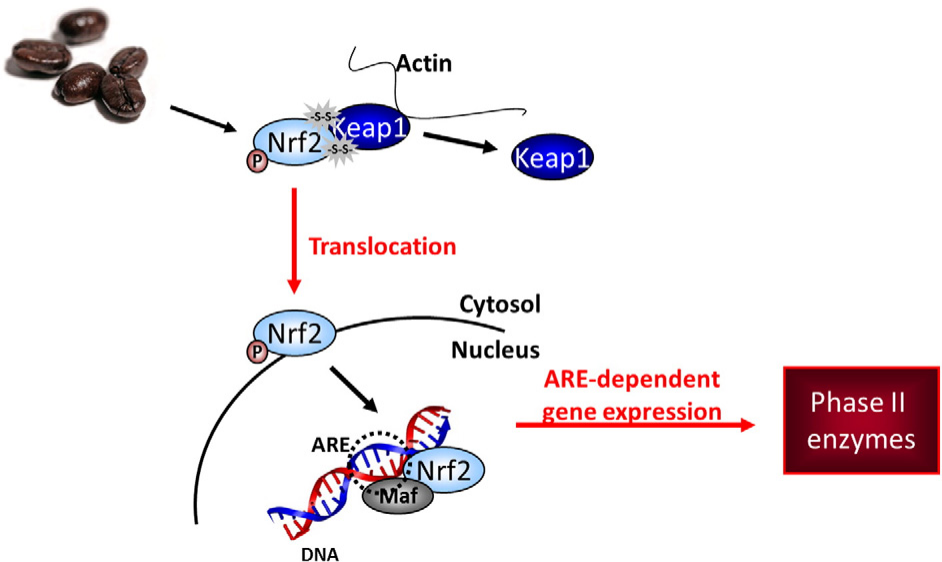
Schematic describing induction of the Nrf2/ARE-detoxifying pathway by coffee, influenced by the genetic background of the individual.

Although the capacity to measure the genome, proteome, and perhaps soon the metabolome has largely developed during the last few years, advances in the associated methodology for measuring functional and behavioral components of the nutritional phenotype are not yet fully developed. We have now demonstrated downstream effects on transcriptional regulation within the antioxidative phase II pathway are dependent upon the genotype of the key transcriptional regulator Nrf2 (Fig. 4). We therefore suggest that genotype must be considered when determining criteria to evaluate treatment and outcome strategies in human antioxidant interventions and therapies. Specifically, genotype needs to be considered when targeting Nrf2-dependent gene response in order to estimate the chemopreventive potency of bioactive food/food constituents.

## Experimental

### Coffee brew

The study coffee brew consisted of a special roasted and blended Arabica coffee, characterized by a high concentration of both, green and roasted bean constituents, especially n-chlorogenic acid (252 mg/L of a total of 817 mg/L of all chlorogenic acids) and NMP (128 mg/L). The caffeine level (558 mg/L) was in the average range of conventional coffee brews. The ground coffee was delivered in vacuum bags (12.5 g each). Immediately before consumption, the coffee brew was freshly prepared using a Senseo coffee maker (Philips). Each individual consumed 250 mL of the coffee three times a day.

### Subjects

The study was approved by the University Human Research Ethics Committee (Griffith University approval number EC00162) with participants providing written consent to participate in the study. Signatures were obtained in the presence of an independent witness and the appropriate number of participants estimated by a sample size calculation to determine power a priori (Boettler et al., 2012). The final test population was comprised of 25 male and 26 female (total n = 51), healthy non-smoking (age 18–61, BMI 19–38) Caucasians. Originally, 57 individuals were recruited, 6 individuals did not complete the trial for various reasons (gastric symptoms or sleeplessness after coffee consumption). All participants were regular coffee drinkers (2–6 cups of coffee/day). Participants were asked to maintain their usual dietary habits for the duration of the study, except for the coffee intake, caffeinated products, dietary supplements and foods rich in polyphenols. Two detailed nutritional information bulletins were provided to each participant of the trial. Bulletin no. 1 defined polyphenol-free beverages, offering examples and described their consumption. Bulletin no. 2 provided general nutritional information and a detailed list of different foods including fruits, vegetable and other food categorized as “permitted” and “permitted in minor amounts”. All volunteers were informed of the objectives of the study and consent was received for their participation. Exclusion criteria included smoking and the use of medication. In addition, competitive athletes were excluded. The 8-week intervention trial was designed as follows: weeks 1–2, 1st wash-out; weeks 3–6, coffee consumption and weeks 7–8, 2nd wash-out. During the consumption period, participants consumed 750 mL of freshly brewed coffee (with/without sugar, addition of milk up to 50 mL) in three equal portions (morning, noontime, afternoon). During the two wash-out periods, the coffee brew was replaced by equal volumes of water. Nutritional reports covering a 7-day period were completed by participants in the last week of each study period. Urine/blood sampling was performed at the beginning of the study and on the last day of each study period in the morning, after a fasting period of at least 6 h.

### Genomic DNA isolation

For genomic DNA (gDNA) isolation, the QIAamp DNA Mini kit (QIAGEN) was used and gDNA purified as per manufacturer's instructions. The final concentration of DNA was quantified using a ND-1000 spectrophotometer (Nanodrop, Delaware).

### Sequencing

#### SNPs rs35652124, rs6706649 and rs6721961

To obtain the 424 bp amplicon of the polymorphic region of the NFE2L2 gene, 80 ng of gDNA was amplified with 4 μL of GoTaq® Flexi buffer (Promega), 2.5 mM MgCl_2_, 0.2 mM dNTP, 0.2 μM of each NFE2L2 primer (forward primer 5′–GACCACTCTCCGACCTAAAGG–3′ and reverse primer 5′–CGAGATAAAGAGTTGTTTGCGAA-3′) and 0.2 μL GoTaq® to a total reaction volume of 20 μL. Amplification was performed on a Veriti™ 96-well Thermal Cycler (Applied Biosystems) with an initial step of 94 °C × 10 min, 30 cycles of 94 °C × 45 s, 55 °C × 45 s and 72 °C × 45 s and a final extension of 72 °C × 7 min. 5 μL of PCR product was electrophoresed on 2% agarose gels stained with ethidium bromide for 30 min at 80 V. PCR products were purified with the ExoSAP-IT® PCR cleanup kit (Affymetrix/USB) according to manufacturer's instructions. DNA concentration was quantified using a ND-1000 spectrophotometer (Nanodrop, Delaware) and diluted to a concentration of 20 ng/μL DNA. For the sequencing reaction 1 μL of DNA was added to 5 μL of BigDye Terminator v3 (BDT v3.1, Applied Biosystems), 1.3 μL of each NFE2L2 primer and 3.0 μL of 5 × BDT v3.1 Sequencing buffer. Amplification cycles were as follows: 96 °C × 1 min, 30 cycles of 96 °C × 10 s, 50 °C × 5 s and 60 °C × 4 min followed by 4 °C × 5 min, 10 °C × 5 min and 4 °C × 2 min. The product was transferred to a 1.5 mL tube and ethanol precipitated by adding 2 μL of iced cold 3 M sodium acetate (pH 5.2) and 2 μL of 125 mM EDTA (pH 8.0). The sample was vortexed, centrifuged at 10,000 *g* for 5 min, followed by the addition of 50 μL of 100% ethanol followed by repeated vortexing and re-spinning the samples at 10,000 *g* for 5 min. Samples were then incubated for 15 min (RT), precipitated by centrifugation at 10,000 *g* followed by incubation at 4 °C for 20 min. The pellet was then rinsed in 70% ethanol, vortexed and centrifuged at 10,000 *g* at 4 °C for 5 min. The pellet was dried using a DNA Speed Vac® (Savant) on high drying mode for 5 min. Samples were then resuspended in 15 μL dH_2_O. The purified products were directly added into a Micro Amp™ optical 96 well reaction plate (Applied Biosystems) to be analyzed on a 3130 genetic analyzer (Applied Biosystems).

### GST1A1 genotyping

GST1A1 genotyping was carried out by RFLP analysis. For PCR amplification, gene-specific primers (forward primer 5′-TGTTGATTGTTTGCCTGAAATT-3′; reverse primer 5′-GTTAACGCTGTCACCGTCCT-3′) were used to generate a 480 bp PCR product spanning the polymorphism. For the amplification of GSTA1 40 ng of gDNA was amplified with 4 μL of GoTag® Flexi buffer (Promega), 2.5 mM MgCl_2_, 0.2 mM each dNTP, 0.1 μM of each GST1A1 primer and 1U Taq polymerase added. Amplification was performed on a VeritiTM 96-well Thermal Cycler (Applied Biosystems) with an initial denaturation of 94 °C × 10 min, 30 cycles of 94 °C × 45 s, 58 °C × 45 s and 72 °C × 45 s, and a final extension of 72 °C × 7 min. PCR products were electrophoresed on 2% agarose gels stained with ethidium bromide for 30 min at 80 V. A no template control was included to detect possible contamination issues. This PCR product was then digested with EAR1 (New England Biolabs, Beverly, Massachusetts, USA). PCR reaction products were digested using 4 U of restriction enzyme *Ear1*, 2 μL of 10 × Buffer and 18 μL PCR product for 5 h at 37 °C. A no template control was included. The digested products were resolved on a 4% ultrapure agarose gel post-stained with 20 μL ethidium bromide for 10 min. The GST1A1*A genotype produced a band at 480 bp. A heterozygote GST1A1*A/B resulted in three bands at 480, 380 and 100 bp and the GST1A1*B genotype resulted in two bands at 380 and 100 bp.

### UGT1A1 genotyping by size fragment analysis

First, PCR was performed using one unlabelled and one 5′-end 6-FAM labeled primer (forward primer: 5′-156-FAM/AAGTGAACTCCCTGCTACCTT-3′, reverse primer 5′-CCACTGGGATCAACAGTATCT-3′) resulting in a 253 bp PCR product, flanking the polymorphic TA locus in the promoter region. Briefly, 80 ng of gDNA was amplified with 4 μL of a GoTag® Flexi buffer (Promega), 2.5 mM MgCl_2_, 0.2 mM each dNTP, and 0.1 μM of each UGT1A1 primer. 1 U Taq polymerase was added for a total reaction volume of 20 μL. Amplification was performed on a VeritiTM 96-well Thermal Cycler (Applied Biosystems) with an initial denaturation of 94 °C × 15 min, followed by 30 cycles of 95 °C × 30 s, 58 °C × 40 s and 72 °C × 40 s, followed by a final extension of 72 °C for 7 min. Following amplification, PCR products were electrophoresed on 2% agarose gels stained with ethidium bromide for 30 min at 80 V. A no template control was included to detect any contamination. For sequencing, 0.5 μL of PCR product was administered to 0.25 μL 1:10 diluted GeneScan™ 500 LIZ Size Standard (Applied Biosystems) and 9.25 μL of HiDi formamide (Applied Biosystems). Samples were diluted 1:5, subjected to sequencing on the 3130 genetic analyzer (Applied Biosystems) for (TA)_n_, and scored via Gene Mapper v4.0 software (Applied Biosystems). Control DNAs from previously sequenced individuals known to have a 6/6, 6/7 or 7/7 genotype were included in the PCR analysis. The amplified product yielded a 93-or a 95-base pair fragment, which corresponded to (TA)_6_ and (TA)_7_, respectively.

### Gene transcription by Q-PCR

At each of the four different BC points, venous blood samples were collected in EDTA-tubes and stored at RT until the sampling period was completed. RNA was then extracted from the human PBLs, and Q-PCR performed as previously reported (Boettler et al., 2012). Total RNA was extracted from isolated PBLs following manufacturer's handbook of the RNeasy® Mini Kit (QIAGEN, Hilden, Germany). Following this, 2 μg RNA was reverse-transcribed using Oligo-dT primers and the Omniscript® Reverse Transcription Kit (QIAGEN, Hilden, Germany). cDNA obtained from the RT reaction (amount corresponding to 2 μg of total RNA) was subjected to Q-PCR using QuantiTect SYBR® Green PCR (QIAGEN, Hilden, Germany). The primer assays used were: Hs_NFE2L2_1_SG, QT00027384. ß-Actin: Hs_ACTB_1_SG, QT00095431 GST1A1: Hs_GST1A1_1_SG, QT00060739. GSTT1: Hs_GSTT1_2_SG, QT010751638. UGT1A1: Hs_UGT1A1_1_SG, QT00020860 (QIAGEN, Hilden, Germany). Primer concentrations and Q-PCR reaction parameters were according to manufacturer's guidelines in QuantiTect SYBR® Green PCR Handbook 11/2005 (QIAGEN, Hilden, Germany). Each sample was amplified in triplicate. A no RevT control was included for all assays.

### Statistical analysis

The fold changes in expression of the target gene relative to the internal control gene (ß-actin) was analyzed using Bio-Opticon Software and the *C*_T_ data was imported into Microsoft Excel 03. Data of all assays was analyzed by the 2^− ΔΔ*C*^_T_ method. ß-actin was chosen as reference gene due to its higher stability with respect to the relative transcription levels determined and its non-influenceability during the different antioxidative status levels of the PBLs compared to GAPDH (glyceraldehyde 3-phosphate dehydrogenase) and RPL13 (ribosomale protein L13a) as reference genes during preliminary testings. Data presented are the mean ± SD of individuals, grouped according to their respective genotypes and presented as relative transcription of wash-out (BC1 or BC2) = 1. Indicated significant differences in relative transcription levels were calculated by a paired Student's t-test on the relative expression values of each sample and indicate *p < 0.05 and **p < 0.01. The assumption of normality, underlying the t-tests was verified by the Shapiro–Wilk test.

In order to investigate the impact of Nrf2, GST1A1 and UGT1A1 polymorphisms, participants were stratified into seven or three subgroups, respectively according to their genotype: Nrf2: WT (group 1, N = 9), only − 617 (group 2, N = 2), only − 651 (group 3, N = 4), only − 653 (group 4, N = 16), − 617 + − 653 polymorphisms (group 5, N = 7), − 651 + − 653 polymorphisms (group 6, N = 9) and − 617 + − 651 polymorphisms (group 7, N = 2). GST1A1: A/A (group 1, N = 24), A/B (group 2, N = 18) and B/B (group 3, N = 9); UGT1A1: [TA]_6/6_ (group 1, N = 20), [TA]_6/7_ (group 2, N = 18) and [TA]_7/7_ (group 3, N = 6). Groups of Nrf2, GST1A1 and UGT1A1 polymorphisms were grouped according to Nrf2, GST1A1 and UGT1A1 based on absolute height of measurements at each BC as well as pairwise differences between BC's. Groups were compared pairwise by Wilcoxon rank sum tests.

Statistical hypotheses were tested at the 5% level of significance against a two-sided alternative. Calculations were done by the statistical software SPSS.

## Figures and Tables

**Table 1:** Genotype distribution of Nrf2, GST1A1 and UGT1A1 in the participants of the coffee intervention study.

Gene	SNP	Frequency		Literature		Ref.
N	[%]	n	[%]
Nrf2	− 617C/C (WT)	39/49	80	16/20	80	Marzec et al. (2007)
− 617C/A	10/49	20	4/20	20	Marzec et al. (2007)
− 651G/G (WT)	34/49	69	18/20	90	Marzec et al. (2007)
− 651G/A	15/49	31	2/20	10	Marzec et al. (2007)
− 653A/A (WT)	18/49	37	15/20	75	Marzec et al. (2007)
− 653A/G	31/49	63	5/20	25	Marzec et al. (2007)
GST1A1	A/A (WT)	24/48	50	106/27	38	Coles et al. (2001)
A/B	18/48	38	133/27	48	Coles et al. (2001)
B/B	6/48	12	39/278	14	Coles et al. (2001)
UGT1A1	[TA]_6_TAA (WT)	21/46	46	183/399	46	Van der Logt et al. (2004)
[TA]_6/7_TAA	19/46	41	169/399	42	Van der Logt et al. (2004)
[TA]_7_TAA	6/46	13	47/399	12	Van der Logt et al. (2004)
